# The evaluation of a fourth-generation multi-theory model (MTM) based intervention to initiate and sustain physical activity

**DOI:** 10.15171/hpp.2019.02

**Published:** 2019-01-23

**Authors:** Traci Hayes, Manoj Sharma, Mohammad Shahbazi, Jung Hye Sung, Russell Bennett, Jacqueline Reese-Smith

**Affiliations:** ^1^Behavioral & Environmental Health, School of Public Health, Jackson State University, Jackson, MS, USA; ^2^Department of Psychology, Jackson State University, Jackson, MS, USA

**Keywords:** Physical activity, Exercise, Women, Methods, Prevention and control, Statistical and numerical data

## Abstract

**Background: ** The United States Department of Health and Human Services (USDHHS)recommends that adults achieve 150 minutes per week of moderate-intensity aerobic activity.Most African American women do not meet these guidelines. The purpose of this study was to determine the efficacy of an intervention based on the fourth generation, multi-theory model (MTM) of health behavior change for initiating and sustaining physical activity among African American women when compared to a first generation, knowledge-based intervention.

**Methods: ** The randomized controlled trial (RCT) utilized a pre-test, post-test and 6-week followup evaluation with an experimental (n=25) group and a comparison group (n=23). Process evaluation for satisfaction and program fidelity was conducted along with impact evaluation for changes in MTM constructs, intent to initiate and sustain physical activity, minutes of physical activity, body mass index (BMI), waist circumference and blood pressure in hypertensives.

**Results:**The MTM-based intervention proved significantly efficacious in increasing the minutes of physical activity from pre-test mean of 37 minutes to 172 minutes at follow-up (mean difference135.08 minutes, 95% CI: 106.04 to 164.13, P<0.0001), reducing waist circumference from pretest mean of 39 inches to 38 inches at follow-up (mean difference -1.12 inches, 95% CI: -1.70 to-0.545, P<0.001) and modifying the MTM construct of changes in physical environment from a mean of 7 units at pre-test to 9 units at follow-up (mean difference 2.08 units, 95% CI: 0.73 to 3.43, P<0.004) when compared to the knowledge-based intervention over time.

**Conclusion:** There were directional improvements in the mean scores for most of the study variables over time for the MTM intervention group and statistically significant improvement in minutes of physical activity and waist circumference.

## Introduction

Being physically active is recognized as one of the most significant behaviors that can positively impact overall health and well-being.^1-3^ Physical activity (PA) is defined by the World Health Organization (WHO) as “bodily movements produced by the skeletal muscles that require energy expenditure”.^4^ PA can be further categorized as light, moderate and heavy intensity. Low intensity activities such as standing, or strolling are referred to as basal activities or inactivity.^3,5^ Health benefits are not achieved at this level.^3^

In 2008, a joint effort of the US Department of Health and Human Services (USDHHS) and the US Department of Agriculture (USDA) established the PA guidelines for the nation and recommended that all US adults achieve 30 minutes a day of moderate intensity aerobic PA or 150 minutes per week, along with muscle-strength training 2 to 3 times per week. Engaging in moderate-intensity PA such as dancing, running, brisk walking, moving consistently for at least 150 minutes per week, offers wide-ranging health advantages such as reducing the risk of coronary heart disease, type 2 diabetes, breast cancer, colon cancer, and premature death.^5^ PA is associated with lowering blood pressure among hypertensives, reducing obesity and providing numerous psychological advantages.^5^

The WHO identified the lack of PA as the fourth leading risk factor for world-wide mortality.^6^ Unfortunately, many Americans (51%) are not meeting the daily recommended levels of PA.^7,8^ Among the various subgroups, African American women are the subgroup most likely to be inactive and most susceptible to the health problems associated with low PA levels.^9-13^ Numerous studies have been conducted to better understand factors that prevent and promote PA among African American women. Researchers have used in-depth semi-structured interviews, objective assessment of PA and photovoice elicitation to probe the factors that move elderly African-American women’s decision to exercise.^14^ The study identified older African American women’s lack of knowledge about PA, unsafe walking environments and crime, stressors like racism and discrimination, and economic constraints lowered the likelihood of exercising among older African American women. Bryant Smalley et al sought to understand how racial identity influenced African American women’s interest in engaging in weight loss and exercise through a cross-sectional study measuring ethnical identity and willingness to change behavior based on the transtheoretical model.^11^

Interventions to increase PA among African American women have varied from home-based to community mass-education-focused programs.^15^ Schoeny et al conducted a cluster randomized control trial to improve PA among African American women.^16^ Testing 3 conditions: group discussion with personal call, group discussion with automated call and group discussion only, the researchers found that group with personal calls increased steps per day when compared to other groups.^16^ In another study, treatments focused on participants receiving support from different sources during a 10-week walking program. By the completion of the program, about 71% of the study participants met or surpassed their step goals.^17^

Many researchers suggest using theories to improve understanding of behavior and to craft interventions for the maintenance of the changed behavior.^18-20^ Several PA interventions have relied upon popular theories to promote PA among African American women.^21-25^ Each study acknowledged the benefits of utilizing a theoretical framework to drive the development and deployment of their intervention to promote and increase PA. However, there is further need to ameliorate theoretical approaches.

The first generation approaches in health education were the knowledge-based programs. These were followed by second generation skill-based programs and then single theory third generation programs. The latest fourth generation trend is to use multiple theories that use technology and deliver precision interventions.^26,27^ A fourth-generation approach, the multi-theory model (MTM) of health behavior change, layers varied concepts pertaining to internal and external mediators to modify one’s health behavior for long-term implementation. The MTM addresses the initiation and the sustenance of the behavior. Under MTM, the initiation of behavior change includes 3 constructs: participatory dialogue (when advantages outweigh the disadvantages), behavioral confidence (belief in one’s capacity to achieve the desired behavior) and changes in physical environment (surroundings that enable behavior implementation).^26,27^ The sustenance of behavior change includes emotional transformation (overcoming self-doubting intentions), practice for change (evaluating and adjusting efforts to achieve desired behavior) and changes in social environment (leveraging positive relationship to achieve the desired behavior).^26,27^ The purpose of the study was to determine the efficacy of an intervention based on the MTM for health behavior change for initiating and sustaining PA among African American women. PA for this study is defined as 30 minutes or more of moderate-intensity aerobic movement and does not focus on the requirement for muscle-strengthening.

## Material and Methods

### Study design, population and sampling

This randomized-controlled study utilized, a pre-test, post-test and follow-up evaluation with 2 arms: experimental arm and a comparison arm. Participants who met the inclusion criteria of not having been physically active for 150 minutes or more during the previous seven days were randomly assigned to the 2 groups by applying the random assignment method of approximately 50% using the Statistical Package for the Social Sciences (SPSS). The comparison group received a knowledge-only approach that utilized a didactic style to disseminate PA benefits and information to the participants during the 3 60-minute sessions. The comparison group was not exposed to the multi-component, interactive approach which is the foundation of the MTM, a fourth-generation approach. The measurable variables included the 6 theory constructs: 1) participatory dialogue, 2) behavioral confidence, 3) changes in physical environment, 4) emotional transformation, 5) practice for change, and 6) changes in social environment; along with the participants’ intent to initiate PA and intent to sustain PA, frequency of minutes of aerobic PA, and anthropometric and clinical measures (waist circumference, body mass index [BMI] based on weight and height) and systolic and diastolic blood pressure (among hypertensives).

### Participants 

Adult women, ages 18-69 years, of African American or of African ancestry, residing in the Central Mississippi region were recruited via radio, public access television, social media and word-of-mouth to participate in the study. A minimum sample size of 20 was obtained using G*Power with an alpha of 0.05, a power (1-beta) of 0.80, an effect size f=0.30 (medium) for 2 groups for the outcome variable of PA minutes. The sample requirement was increased by 20% to 24 participants per group to account for any attrition. This intervention was conducted between January 2018 and June 2018.

### Instrumentation

A self-report instrument, ratified for content and construct validity and internal consistency in a prior study, was used to measure the constructs.^28^ The women completed the survey and were measured for weight/height, waist circumference, and blood pressure at the pre-test (program orientation), post-test (session 3, three-weeks after) and the follow-up (6-weeks after the post-test) session.

The initiation model was established with 3 theory constructs: participatory dialogue, behavioral confidence and change in physical environment. The difference in participants’ views of the advantages of PA as greater than the disadvantages of engaging in PA is participatory dialogue. Five questions on a scale of 0-4, with 0 being “never” to 4 = “always”, were used to assess the advantages and disadvantages components of participatory dialogue. The range for both advantages and disadvantages was 0 to 20 units. There is increased likelihood of initiating PA when the score is higher for advantages than disadvantages. Maintaining some similarities to self-efficacy, the construct, behavioral confidence represents the certainty that the participants would engage in PA. Behavioral confidence range was 0 to 20 units. There was increased likelihood of PA behavior change when the score was higher. Three questions helped to identify the likelihood that the change in physical environment would result in the participants’ change in behavior from physically inactive to active. The responses were scored from not at all sure (=0) to completely sure (=4). The possible range was 0 to 12 units; a higher score depicts the likelihood to have behavior change from inactive to active. The likelihood to initiate PA was determined by participants responses to, “How likely is it that you will increase your aerobic PA to 150 minutes in the upcoming week?”, along with the previous 18 questions for the 3 initiation constructs.

The sustenance model was established with 3 theory constructs: emotional transformation, practice for change and change in social environment. The construct of emotional transformation was assessed using 3 questions. The choices ranged from not sure at all (0) to completely sure (4). A high score indicated a likelihood of sustenance PA behavior change. The participants’ likelihood to sustain PA behavior based on change in social environment was assessed using 3 questions. The final question, “How likely is it that you will increase your aerobic PA to 150 minutes every week from now on?”, assessed sustenance or long-term implementation of aerobic PA.

BMI was calculated by entering height (m) and weight (kg) into an online BMI generator.^29^ A normal BMI for adult women is 18.5–24.9 kg/m^2^.^30,31^ Hypertensive blood pressure was considered as a systolic blood pressure of 130 millimeters of mercury (mm Hg) or greater and diastolic blood pressure as 90 mm Hg or greater. Blood pressure was recorded using the Omron 5 series (HEM-7131-Z).

### Interventions

**Figure 1:**
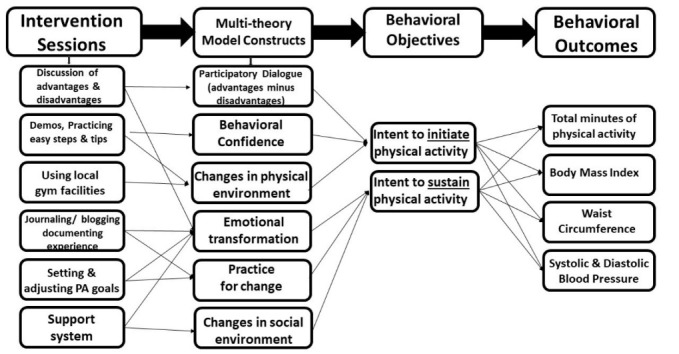
Physical activity intervention logic diagram.

The study orientation and enrollment along with the interventional sessions and 6-week follow-up were conducted at a recreational center on the campus of a midsize Historically Black University in the Southern United States. The participants in the experimental arm (n=25) engaged in activities based on the fourth generation MTM constructs to influence behavior adoption and behavior sustenance. The MTM-based study participants attended three 60-minute sessions over a 3-week period. The MTM intervention was highly interactive, consisting of bi-directional conversations about the advantages and disadvantages of PA, participating in demonstrations of moderate-impact exercises and movements, 10 to 15 minutes workout sessions, instruction on gym equipment, affective learning activities, participation with regard to self-monitoring of the behavior and mobilization of social support. The study participants in the comparison arm received standard first-generation knowledge-based instruction (n=23). The comparison intervention relied on a didactic approach consisting of lectures and Power Point presentations. The experimental and comparison sessions were monitored by an independent observer who verified content and recorded class times to ensure compliance with the study protocol. Participants completed a satisfaction survey during the follow-up session. Figure 1 depicts the intervention logic diagram.

### Statistical Analyses

All data were analyzed using IBM SPSS version 25. Descriptive statistics for sociodemographic variables (age, employment and income) and the MTM constructs were conducted and are presented in Table 1. Chi square test compared the differences between categorical variables such as education and employment for the experimental and comparison groups. Two-tailed *t* test was be used to detect any baseline differences in age between the groups. Means scores over 3-time points for the same individuals were measured using repeated measures analysis of variance (ANOVA) test. Sphericity assumed within subject effects tested using the Mauchly’s test for sphericity were used. The significance level (α) was set at *P *< 0.05. Repeated measures ANOVA was conducted to discern statistically significant differences in the means (from before to after to 6-week follow-up in the interventions) for each theory construct, minutes of PA, and the anthropometric and clinical measures between the experimental (based on MTM) and comparison (knowledge-based) groups. Repeated measures analysis of covariance (ANCOVA) was applied for covariates of participatory dialogue and emotional transformation which were found to be significantly different between the 2 groups at pre-test.

## Results

**Figure 2:**
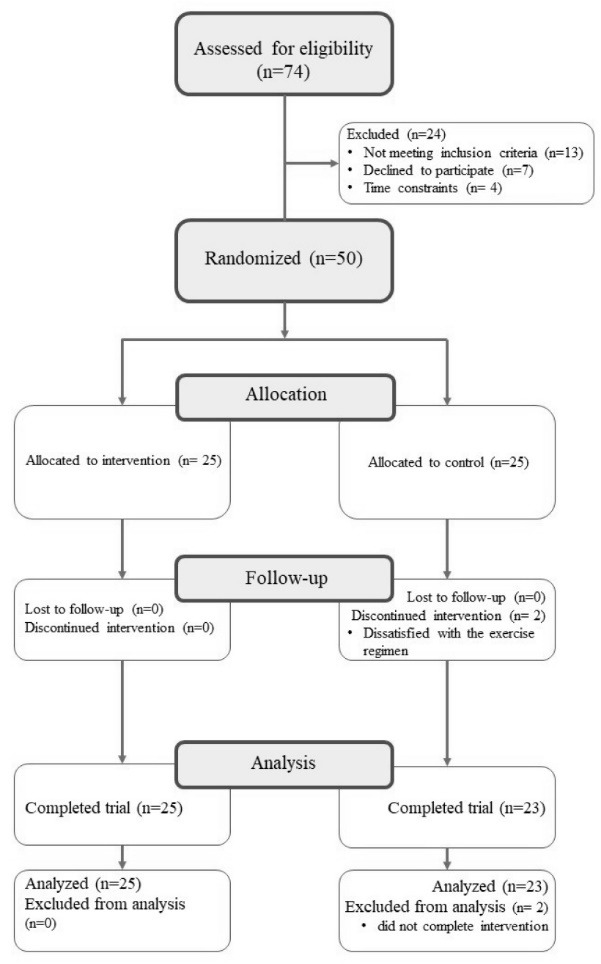
CONSORT flow diagram. Participants flow through the study and reasons for dropout.

A total of 50 women primarily from Central Mississippi were recruited for this study. The CONSORT diagram in Figure 2 shows the screening, randomization, and follow-up of the participants.

Twenty-five (n = 25) participants completed the experimental arm of the intervention. There was an attrition of 2 participants, so the comparison group was comprised of 23 participants (n = 23). Intention-to-treat (ITT) analysis was not possible as 2 participants from comparison group were lost to follow-up and outcome data could not be obtained from them. Since the loss was very small and ITT is controversial due to use of data from non-compliant participants it was decided to exclude the 2 participants from the analysis. The comparisons of the demographic characteristics of the experimental and comparison groups at pretest are depicted in Table 1. The female participants in the experimental and comparison groups did not statistically differ in any of the demographic variables. All participants were African-American women. More than 80% of the participants had some college education or more. There was no statistical difference in experimental group’s mean age (48.52 years) and the comparison group (46.57 years).

More than half of the experimental group (n = 15) and the comparison group (n = 18) participants were employed. There were no significant differences in demographic variables at pretest.

The experimental group and comparison group evaluated the intervention based on the content of the session, appropriateness of the content, session activities, facilitation by the health educator, pace of the sessions, perceived learning from the sessions, and usefulness of the materials. More than 80% of the participants in the experimental group regarded the program as very good and excellent and 100% responded that they would be willing to participate in future research. Regarding program fidelity both the experimental group and comparison groups adhered to the protocols revealing that the programs were delivered in the manner they were designed. There was no significant difference in the mean times for the sessions between the experimental and comparison groups (*P *> 0.05) indicating equivalence of dose in both the groups.

The summary of the descriptive statistics for the various construct subscales as well as the anthropometric (BMI, weight, height and waist circumference) and clinical measures (systolic and diastolic blood pressure) are also reported in Table 2. There was no significant difference in the subscales (advantages of PA, disadvantages of PA, behavioral confidence, change in physical environment, emotional transformation, practice for change and change in social environment) between the experimental group and the comparison group at pretest. There was a significant difference among participatory dialogue (advantages minus disadvantages) (*P *= 0.04), and emotional transformation (*P *= 0.01) at pretest. The mean score for experimental group was higher by approximately 3 units for participatory dialogue and by 2 units for emotional transformation compared to the comparison group. These 2 variables were used as covariates and adjusted through use of repeated measures ANCOVA for significant variables. The results of the ANCOVA did not alter the previous results.

In Table 3, the mean score for the intent to initiate PA increased at pre-test and follow-up for the experimental group. The experimental group mean in minutes of PA increased by 135 minutes to almost 173 minutes. The mean in minutes of PA for the comparison group increased at post-test and follow-up but did not meet the PA recommendation to participate in moderate to vigorous aerobic activity for a minimum of 30 minutes per day for 5 days or 150 minutes a week.^31^ There was an increase in the mean for intent to sustain PA among the experimental group though not statistically significant. The mean for hypertensive systolic and diastolic blood pressure at pre-test and the 6-week follow-up decreased for the experimental group, but were not statistically significant. Aside from the changes in social environment construct, the other 5 constructs, minutes of PA, BMI, and blood pressure improved for the experimental group. The statistically significant variables included minutes of PA, change in physical environment construct and waist circumference.

### Salient findings

The data recorded at pre-test, post-test and follow-up were analyzed using repeated measures ANOVA. There was a significant change in group means for 3 variables: minutes of PA, changes in physical environment construct, and waist circumference between the MTM-based group compared to the comparison (knowledge-based) group.

### Minutes of PA

**Figure 3:**
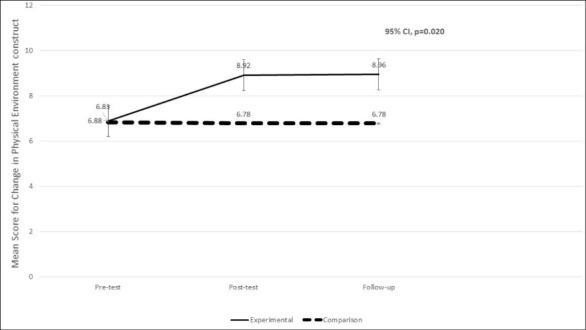
Changes in mean minutes of physical activity from pre-test, post-test to follow-up for experimental and comparison groups.

The minutes of PA were captured for the previous seven days or prior week. In repeated measures ANOVA, Mauchly’s test of sphericity was not significant (*P *= 0.95) and in the test of within-subjects effects, a significant interaction was found. The interaction for the group x time for the mean number of PA minutes was statistically significant, with a *P* value of 0.0001. Less than alpha (α = 0.05), implies a difference in the minutes in PA mean score for the experimental group when compared to the comparison group at pre-test, post-test and follow-up. The mean for change in minutes of PA for the MTM-based group increased from 37.20 minutes at pre-test to 172.28 minutes at the 6-week follow-up. This is an increase of 135 minutes allowing the participants to meet the daily recommended level. The comparison group increased from 41.96 minutes to 98 minutes at the 6-week follow-up but did not meet the recommended daily minutes of PA. The cell mean graph for the changes in mean minutes of PA from pre-test, post-test to follow-up for experimental and comparison groups is depicted in Figure 3.

### Changes in Physical Environment Construct

**Figure 4:**
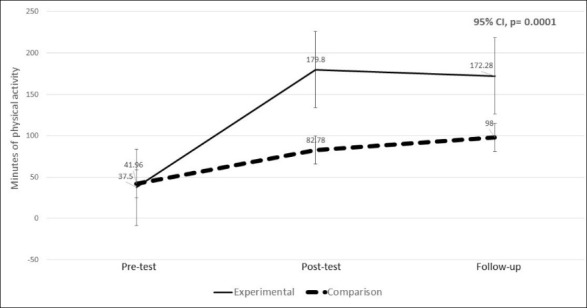
Changes in mean scores for MTM construct of changes in physical environment from pre-test, post-test to follow-up for experimental and comparison groups.

Utilizing repeated measures ANOVA, the Mauchly’s test of sphericity was not significant (*P *= 0.43) and in the test of within-subjects effect was statistically significant. There was a significant difference (*P *= 0.020) in the mean for changes in physical environment construct for the experimental group when compared to the comparison group at pre-test, post-test, and 6-week follow-up. The mean for change in physical environment for the experimental group increased from 6.88 units at pre-test to 8.96 units at the 6-week follow-up. This is an increase of 2.08 units. The comparison group decreased from 6.83 units to 6.78 units at the 6-week follow-up. The results are shown in Table 3 and the cell mean graph for changes in mean scores for the MTM construct of changes in physical environment from pre-test, post-test to follow-up for experimental and comparison groups is depicted in Figure 4.

### Waist Circumference 

**Figure 5:**
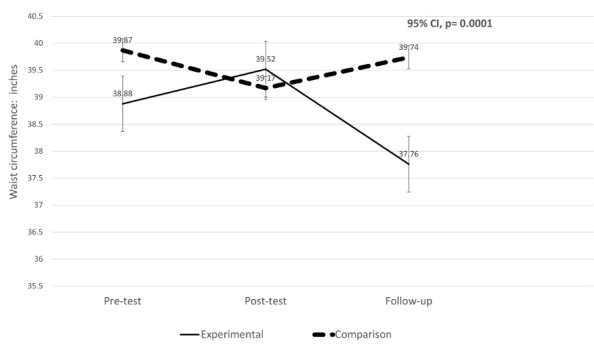
Changes in mean waist circumference from pre-test, post-test to follow-up for experimental and comparison groups.

Normal waist circumference is accepted as less than 35 inches for women.^30,31^ Tested in the within subjects group x time interaction term, the mean for waist circumference was statistically significant (*P *= 0.0001). The Mauchly’s test of sphericity was not significant (*P *= 0.37). The MTM-based experimental group mean for waist circumference was different than the comparison group at pre-, post-test and follow-up.^30^ The mean for waist circumference for the experimental group decreased from 38.88 inches at pre-test to 37.76 inches at the 6-week follow-up. This is a decrease of 1.12 inches. The comparison group decreased insignificantly, from 39.87 inches to 39.74 at the 6-week follow-up. The results are presented in Table 3. Figure 5 depicts the cell mean graph for changes in the mean waist circumference from pre-test, post-test to follow-up for experimental and comparison groups.

The MTM-based participants experienced directional improvement in 11 of the twelve variables; such movement was not true for the knowledge-based comparison group.

## Discussion

The purpose of the study was to determine the efficacy of an intervention based on the MTM for health behavior change for initiating and sustaining PA among African American women. The primary objective of this study was to compare the changes in variable group means in the experimental (fourth generation MTM-based) intervention with those in the comparison (first generation knowledge-based) intervention at pre-test, post-test and 6-week follow-up. Three of the 12 measures were statistically significant: 1) the minutes of PA, 2) changes in physical environment construct, and 3) waist circumference. The MTM-based experimental group experienced directional improvements in 11 of the 12 variables.

In this study, the minutes of PA was gathered through a self-report. The MTM-based experimental group’s minutes of PA mean score increased from pretest to post-test and follow-up. The experimental group increased the minutes of PA to the recommended level of 150 minutes of PA a week. The comparison knowledge-only group did not achieve the recommended minutes of PA. The MTM-based participants attaining and sustaining PA levels at the recommended levels as a result of the sessions was a strength of the intervention.

Participants provided input on the likelihood of accessing an exercise facility, utilizing the equipment in a fitness facility and being able to afford a desirable work out location. This MTM construct of changes in physical environment achieved significance between the experimental group and the comparison group at pre-test, post-test and 6-week follow-up (*P *= 0.020). Having access to and frequently being encouraged by the health researchers to utilize the fitness center, before and after sessions and during the week likely helped to realize these improvements.

The waist circumference mean was statistically different from pre-test to post-test and the 6-week follow-up between the experimental group when compared with the comparison group. The reduction in waist circumference could likely be attributed to the MTM-based experimental group’s increase in PA minutes to the recommended daily level of 30 minutes per day.

While the other variables did not result in a significant difference in group means, each variable for the MTM-based experimental group moved in a direction of improvement, suggesting an increased likelihood of participating in PA. Intent to initiate PA and intent to sustain PA group means rose from pre-test to 6-week follow-up for the MTM-based experimental group, this was represented in the increased minutes of PA. The comparison group mean for intent to initiate PA remained the same from pre-test to the 6-week follow-up; and increased slightly for intent to sustain PA, moving from 2.09 to 2.39 units at the follow-up. Hypertensive systolic and diastolic blood pressure decreased from pre-test to 6-week follow-up for both the groups though the change was not statistically significant.

### Limitations

The current study had some limitations. An efficacy study has the potential to overestimate the intervention’s effect when implemented for practice in a clinical setting.^32^ This limitation can be addressed by future replication and effectiveness studies. The study met the sample requirement of 20 participants in each group; however, the sample was relatively small. A larger sample would improve the study power. The sample consisted of only one racial group and one gender. The results may not be generalizable especially across different groups. Therefore, it should be implemented among various racial and ethnic groups as well as with men. Future replication studies and effectiveness trials will have to be undertaken. The study relied on self-reports of PA. The participants recalled and recorded the number of PA minutes from the previous week. Future studies can use objective measures such as the use of accelerometers in recording PA. Finally, the survey brevity may have limited its ability to probe the constructs in their entirety.

### Strengths

The study strengths include demonstrating improvement in means of study variables for the experimental MTM-based group. Importantly, the study showed that MTM-based intervention was successful in sustaining the effects of PA behavior change which is a challenging enigma in our field. The study proved to be an efficacious, brief, and precise intervention that could be administered in community-settings by public health functionaries and could easily be implemented through involvement of physicians and other health care providers in clinical settings.

### Implications for practice

The chief advantage of MTM is that it provides precision interventions which this study was able to demonstrate. This intervention is suitable for replication by public health practitioners in community settings and by health care providers in clinical settings. The content is adaptable for meeting the varying knowledge and experience levels of the participants which allow for easy acceptance by the participants. This study supports the findings from previous research that theory-based interventions provide the best framework for developing health behavior change programs.^1,12,33^ This current study was a fourth-generation intervention, in that, an array of evidence-based strategies was integrated to target short and long-term PA behavior change. The MTM should continue to be reified by replicated efficacy tests and then effectiveness trials.

### Recommendations for future research

It is recommended that the study be replicated for effectiveness with refinements to construct scope in instrumentation. The effectiveness study should address a larger sample, better and expanded instrumentation, longer duration to follow-up, and implementation in multi-centric, real-world settings. Based on the findings of the study, making changes to the instrument include expanding the question items for each construct to mirror the expansive detail given in the sessions. For example, participants should be asked about the benefits of working out at a gym and to identify value added to their lives to further explore participatory dialogue. Behavioral confidence can be expounded upon with questions regarding the sources of confidence or self-belief. Option to increase behavioral confidence were not addressed with the instrument. Monitoring techniques, such as pedometers and mobile apps should be included for the practice for change construct. Relationships with coworkers and religious, community, and civic groups should be explored as sources for support pertaining to the change in social environment construct.

Future research should look at testing the effectiveness of the MTM for health behavior change to better understand how the MTM-based PA program would work in an environment more like the real world.^32^ Such research will help to determine the applicability for MTM in clinics and medical facilities. It is suggested that continued evaluation to identify the combination of MTM strategies that support sustained PA change should be researched.^34^

## Conclusion

This study provides a framework for implementing a fourth-generation intervention to increase PA to the recommended 30 minutes per day for 5 days or 150 minutes per week. The study demonstrated the ability of the MTM-based intervention to successfully increase the minutes of PA, reduce waist circumference and affect significant change in the construct, changes in physical environment. There were directional improvements in the mean scores for most of the study variables over time. This is the first interventional study in which an attempt was made to establish the efficacy of the MTM for health behavior change for affecting change in minutes of PA and improving health outcomes. Future interventions must refine this approach and apply MTM-based interventions in real-world applications.

## Ethical approval

Institutional Review Board (IRB) approval was obtained from Jackson State University (Protocol #0092-17) and informed consent obtained from all participants.

## Competing interests

The authors declare that they have no competing interests.

## Funding

Research Centers in Minority Institutions Translational Research Network (RTRN) under award number U54MD008149.

## Authors’ contributions

TH, MaS, MoS, JHS, RB, and JRS have provided substantial contributions to conception and design, acquisition of data, or analysis and interpretation of data; TH, MaS, MoS, JHS, RB, and Reese-Smith drafted the article or revised it critically for important intellectual content; TH, MaS, MoS, JHS, RB, and JRS gave final approval of the version of the article to be published; and they agree to be accountable for all aspects of the work in ensuring that questions related to the accuracy or integrity of any part of the work are appropriately investigated and resolved.

## Acknowledgements

The authors would like to acknowledge the Research Centers in Minority Institutions Translational Research Network (RTRN) under award number U54MD008149 and participants of the study for their cooperation. Thanks are also extended to the School of Public Health at Jackson State University.

**Table 1:** Summary of the demographic variables distribution: Comparison of experimental (n=25) and comparison groups (n=23)

**Variable**	**Subgroups**	**Experimental** **No. (%)**	**Comparison** **No. (%)**	**Chi-square value**	***df***	***P*** ** value**
Education	High School Graduate	4 (16)	0 (0)	6.62	3	0.09
	Some College	9 (36)	5 (21.7)			
	College Graduate	6 (24)	7 (30.4)			
	Graduate or Professional degree	6 (24)	11 (47.8)			
	Total	25 (100)	23 (100)			
Income	<$40 000	10 (4.2)	10 (45.5)	4.12	3	0.25
	$40 001-$64 999	7 (29.2)	10 (45.5)			
	$65 000 - more	7 (29.2)	2 (9.1)			
	Total	24 (100)	22 (100)			
Employment	Not working	9 (36)	5 (21.7)	2.34	2	0.31
	Working	15 (60)	18 (78.3)			
	Total	24 (100)	23 (100)			
Residential counties	Hinds	17 (68.0)	17 (50.0)	3.92	7	0.79
	Madison	3 (12.0)	3 (13.0)			
	Rankin	1 (4.0)	1 (4.3)			
	Simpson	1 (4.0)	1 (4.3)			
	Jackson	1 (4.0)	0 (0.0)			
	Forest	0 (0.0)	1 (4.3)			
	Shelby, TN	1 (4.0)	0 (0.0)			
Age	Mean (SD)	48.52 (16.55)	46.57 (15.30)	T=0.18	1	0.67

**Table 2:** Means and standard deviations of subscales scores in experimental and comparison groups at pre-test

**Variable**	**Possible range**	**Experimental group** **Mean (SD)**	**Comparison group** **Mean (SD)**	***F***	***df***	***P *** **value**
Constructs						
Participatory dialogue^b^	-20.00 - +20.00	8.84 (5.58)	5.61 (5.22)	4.27	1	0.04
Advantages	0.00 – 20.00	15.48 (4.27)	13.61 (3.37)	2.81	1	0.10
Disadvantages	0.00 – 20.00	6.64 (2.90)	8.0 (2.83)	2.70	1	0.11
Behavioral confidence	0.00 – 20.00	11.84 (5.26)	9.70 (3.87)	2.55	1	0.12
Change in physical environment	0.00 – 12.00	6.88 (3.63)	6.83 (2.85)	0.003	1	0.95
Intention to initiate physical activity	0.00– 4.00	2.84 (1.18)	2.39 (1.03)	1.95	1	0.17
Emotional transformation	0.00 – 12.00	9.04 (2.59)	7.04 (2.70)	6.82	1	0.012
Practice for change	0.00 – 12.00	7.60 (2.72)	6.13 (2.05)	4.40	1	0.42
Change in social environment	0.00 – 12.00	6.20 (3.49)	5.26 (2.72)	1.07	1	0.31
Intention to sustain physical activity	0.00 – 4.00	2.68 (1.07)	2.09 (1.00)	3.93	1	0.053
Minutes of physical activity^a^	150 minutes or more	37.20 (46.85)	41.96 (44.56)	1.29	1	0.72
Anthropometric measurements						
BMI (weight: height ratio)		31.55 (8.49)	34.96 (8.32)	1.96	1	0.17
Waist circumference (inches)		38.88 (6.99)	39.87 (6.48)	0.26	1	0.61
Weight (pounds)	-	191.72 (46.55)	212.70 (66.53)	1.62	1	0.21
Height (inches)	-	65.44 (3.00)	64.78 (3.19)	0.54	1	0.47
Clinical measurements						
Systolic blood pressure (mm Hg)		133 (16.61)	136 (23.33)	0.27	1	0.61
Diastolic blood pressure (mm Hg)		84.96(12.47)	86.70 (15.42)	0.19	1	0.67
Hypertensive systolic blood pressure^c^(mm Hg)	130 or more	143.47 (11.96)	151.85 (18.29)	2.11	1	0.16
Hypertensive diastolic blood pressure^d^(mm Hg)	90 or more	99.25 (10.78)	106.00 (10.41)	1.38	1	0.26

^a^The U.S. Department of Health and Human Services recommended 30 minutes a day for 5 days or 150 minutes per week of moderate to vigorous aerobic physical activity. ^b^Participatory dialogue is calculated by subtracting the disadvantages mean from the advantages mean ^c^The participants were categorized as hypertensive systolic blood pressure (HTSBP), if SBP was measured as 130 mm Hg or greater. ^d^The participants were categorized as hypertensive diastolic blood pressure (HTDBP), if DBP was measured as 90 mm Hg or greater.

**Table 3:** Means and standard deviations of subscale scores in experimental and comparison groups (pre-test/post-test/follow-up)

**Variable**	**Possible range**	**Experimental**			**Comparison**			**Group x Time** ***P*** ** value**
		**Pre-test** **Mean (SD)**	**Post-test** **Mean (SD)**	**Follow-up** **Mean (SD)**	**Pre-test** **Mean (SD)**	**Post-Test** **Mean (SD)**	**Follow-up** **Mean (SD)**	
Constructs								
Participatory dialogue	-20.00–20.00	8.84 (5.58)	8.00 (4.94)	7.52 (4.36)	5.61 5.22)	6.74 (5.39)	5.35 (4.59)	0.42
Advantages	0.00–20.00	15.48 (4.27)	15.16 (3.90)	15.60 (3.04)	13.61 (3.37)	14.74 (3.06)	14.87 (3.04)	
Disadvantages	0.00–20.00	6.64 (2.90)	7.16 (3.06)	8.08 (3.05)	8.0 (2.83)	8.00 (2.83)	8.00 (2.97)	
Behavioral confidence	0.00–20.00	11.84 (5.26)	13.16 (3.53)	14.72 (4.34)	9.70 (3.87)	10.22 (3.66)	10.78 (3.88)	0.31
Change in physical environment	0.00–12.00	6.88 (3.63)	8.92 (2.14)	8.96 (2.81)	6.83 (2.85)	6.78 (3.09)	6.78 (3.19)	0.020
Intent to initiate physical activity	0.00–4.00	2.84 (1.18)	3.04 (0.79)	3.16 (0.90)	2.39 (1.03)	2.17 (0.98)	2.39 (0.89)	0.46
Emotional transformation	0.00–12.00	9.04 (2.59)	8.96 (1.81)	9.68 (2.44)	7.04 (2.70)	6.35 (2.39)	7.09 (2.39)	0.72
Practice for change	0.00–12.00	7.60 (2.72)	7.72 (2.30)	8.28 (3.12)	6.13 (2.05)	5.09 (2.76)	6.48 (2.64)	0.42
Change in social environment	0.00–12.00	6.20 (3.49)	6.44 (3.66)	6.08 (3.15)	5.26 (2.72)	5.30 (2.27)	5.78 (2.54)	0.73
Intent to sustain physical activity	0.00–4.00	2.68 (1.07)	3.00 (1.00)	3.12 (0.93)	2.09 (1.00)	2.09 (1.13)	2.39 (0.78)	0.60
Minutes of physical activity^a^		37.20 (46.85)	179.80 (80.90)	172.28 (67.37)	41.96 (44.56)	82.78 (74.32)	98.00 (94.10)	0.0001
Anthropometric measurements								
BMI (weight: height ratio)		31.55 (8.49)	31.61 (8.50)	31.39 (8.43)	34.96 (8.32)	34.57 (8.93)	35.10 (8.41)	0.16
Waist circumference		38.88 (6.99)	39.52 (6.94)	37.76 (7.22)	39.87 (6.48)	39.17 (6.69)	39.74 (6.82)	0.0001
Weight (pounds)		191.72 (46.55)	191.12 (46.64)	190.56 (46.27)	212.70 (66.53)	210.65 (69.70)	213.52 (67.12)	0.15
Height (inches)		65.44 (3.00)	65.44 (3.19)	65.44 (3.00)	64.78 (3.19)	64.78 (3.19)	64.78 (3.19)	-
Clinical measurements								
Systolic blood pressure		133.00 (16.61)	131.24 (12.69)	137.36 (16.23)	136.00 (23.33)	134.30 (23.67)	133.00 (22.55)	0.17
Diastolic blood pressure		84.96(12.47)	83.92 (11.44)	89.40 (11.28)	86.70 (15.42)	87.61 (14.25)	83.43 (9.02)	0.77
Hypertensive systolic blood pressure (mm Hg)^b^		143.47 (11.96)	135.49 (10.93)	141.13 (10.25)	151.85 (18.29)	143.08 (26.72)	145.08 (22.12)	0.64
Hypertensive diastolic blood pressure (mm Hg)^c^		99.25 (10.78)	92.63 (9.59)	94.25 (9.72)	106.00 (10.41)	100.33 (20.00)	92.00 (4.78)	0.33

^a^The U.S. Department of Health and Human Services recommended 30 minutes a day for 5 days or 150 minutes per week of moderate to vigorous aerobic physical activity. ^b^The participants were categorized as hypertensive systolic blood pressure (HTSBP), if SBP was measured as 130 mm Hg or greater. ^c^The participants were categorized as hypertensive diastolic blood pressure (HTDBP), if DBP was measured as 90 mm Hg or greater. Millimeter of mercury is a manometric unit of pressure.
